# Generic Submanifolds of Nearly Kaehler Manifolds with Certain Parallel Canonical Structure

**DOI:** 10.1155/2014/363429

**Published:** 2014-10-29

**Authors:** Qingqing Zhu, Biaogui Yang

**Affiliations:** School of Mathematics and Computer Science, Fujian Normal University, Fuzhou 350108, China

## Abstract

The class of generic submanifold includes all real hypersurfaces, complex submanifolds, totally real submanifolds, and CR-submanifolds. In this paper we initiate the study of generic submanifolds in a nearly Kaehler manifold from differential geometric point of view. Some fundamental results in this paper will be obtained.

## 1. Introduction

Nearly Kaehler manifolds have been studied intensively in the 1970's by Gray [1]. These nearly Kaehler manifolds are almost Hermitian manifolds with almost complex structure *J* for which the tensor field $\bar{\nabla }J$ is skew-symmetric. In particular, the complex structure is nonintegrable if the manifold is non-Kaehler. As we all know, there are two natual types of submanifolds of nearly Kaehler (or more generally, almost Hermitian) manifold, namely, almost complex and totally real submanifolds. Almost complex submanifolds are submanifolds whose tangent spaces are invariant under *J* and totally real submanifolds are opposite. A well known example is the nearly Kaehler 6-dimensional sphere which has been studied by many authors (see, e.g., [2–7]).

In 1981, Chen introduced preliminary the differential geometry of real submanifolds in a Kaehler manifold ([8]) and gave some basic formulas and definitions. Inspired by that paper, we will generalize some important formulas and proprerties in a Kaehler manifold to a nearly Kaehler manifold. The paper is organized as follows: the basic on nearly Kaehler manifolds and submanifold theory will be recapitulated in Section 2. In Section 3, we give the integrability conditions of the two natural distributions *ℋ* and *ℋ* ^⊥^ associated with a generic submanifold of nearly Kaehler manifold. Finally, we consider generic submanifolds with one of its canonical structures to be parallel. These results enable us to prove the following theorem.

**Theorem 1 .**

Let *M* be a generic submanifold in a nearly Kaehler manifold $\bar{M}$. If *P* (or *F*) is parallel, then the holomorphic distribution *ℋ* is intergrable.

The operator *P* (or *F*) is a canonical structure as the following paper introduced.

## 2. Preliminaries

An almost Hermitian manifold $\left(\bar{M},g,J\right)$ is a manifold endowed with an almost complex structure *J*, that is, compatible with the metric *g*, that is, an endomorphism $J:T\bar{M}\to T\bar{M}$ such that *J*_*P*_^2^ = −*Id* for every $p\in \bar{M}$ and *g*(*JX*, *JY*) = *g*(*X*, *Y*). A nearly Kaehler manifold is an almost Hermitian manifold with the extra condition that the (1,2)-tensor field $G=\bar{\nabla }J$ is skew-symmetric:

(1) $$\begin{array}{c} \left({\bar{\nabla }}_{X}J\right)Y+\left({\bar{\nabla }}_{Y}J\right)X=0, \end{array}$$

for every $X,Y\in T\bar{M}$. Here $\bar{\nabla }$ stands for the Levi-Civita connection of the metric *g*. The tensor field *G* on $\bar{M}$ satisfies the following properties ([1, 2]):

(2) G(X,Y)=−G(Y,X),

(3) G(X,JY)=−JG(X,Y),

(4) g(G(X,Y),Z)=−g(G(X,Z),Y),

(5) g(G(X,Y),Z)=g(G(Y,Z),X)=g(G(Z,X),Y),

where *X*, *Y*, and *Z* are arbitrary vector fields on $\bar{M}$.

We denote the metrics of $\bar{M}$ and its submanifold *M* by the same letter *g*, *TM* is the tangent bundle of *M*, and *T* ^⊥^ *M* is the normal bundle of *M*. If ∇ and ∇^⊥^ denote the Riemannian connection induced on *M* and the connection in the normal bundle *T* ^⊥^ *M*, respectively, then the Gauss and Weingarten formulas are

(6) $$\begin{array}{c} {\bar{\nabla }}_{X}Y=\nabla_{X}Y+\sigma \left(X,Y\right), \end{array}$$

(7) $$\begin{array}{c} {\bar{\nabla }}_{X}\xi =-A_{\xi }X+\nabla_{X}^{⊥}\xi , \end{array}$$

where *X*, *Y* ∈ *TM* and *ξ* ∈ *T* ^⊥^ *M*. The second fundamental form *σ* and the shape operator *A*_*ξ*_ are related to each other by

(8) $$\begin{array}{c} g\left(\sigma \left(X,Y\right),\xi \right)=g\left(A_{\xi }X,Y\right). \end{array}$$

For any vector field *X* tangent to *M*, we put

(9) $$\begin{array}{c} JX=PX+FX, \end{array}$$

where *PX* and *FX* are the tangential and normal components of *JX*, respectively. Then *P* is an endomorphism of the tangent bundle *TM* and *F* is a normal-bundle-valued 1-form on *TM*. For any vector field *ξ* normal to *M*, we put

(10) $$\begin{array}{c} J\xi =t\xi +f\xi , \end{array}$$

where *tξ* and *fξ* are the tangential and normal components of *Jξ*, respectively. Then *fξ* is an endomorphism of the normal bundle *T* ^⊥^ *M* and *t* is a tangent-bundle-valued 1-form on *T* ^⊥^ *M*.

For a submanifold *M* in a nearly Kaehler manifold $\bar{M}$ we define

(11) $$\begin{array}{c} H_{x}=T_{x}M\cap JT_{x}M, \end{array}$$

the holomorphic tangent space of *M* at *x*. *ℋ*_*x*_ is the maximal complex subspace of $T_{x}\bar{M}$ which is contained in *T*_*x*_ *M*.

Similar to [8], we will give several definitions as follows.

**Definition 2 .**

A submanifold *M* in a nearly Kahler manifold (or in an almost complex manifold in general) is called a generic submanifold if dim *ℋ*_*x*_ is constant along *M* and *ℋ*_*x*_ defines a differentiable distribution on *M*, called the holomorphic distribution.

**Definition 3 .**

A generic submanifold *M* in a nearly Kaehler manifold is a totally real (resp., complex) submanifold if *JTM*⊆*T* ^⊥^ *M* (resp., *JTM* = *TM*).

For a generic submanifold *M* in a nearly Kaehler manifold $\bar{M}$, the orthogonal complementary distribution *ℋ* ^⊥^, called the purely real distribution, satisfies

(12) $$\begin{array}{c} H_{x}⊥H_{x}^{⊥},\;PH_{x}^{⊥}\subseteq H_{x}^{⊥},\\ H_{x}^{⊥}\cap JH_{x}^{⊥}=\left\{0\right\}. \end{array}$$

From (9) it is clear that the normal-bundle-valued 1-form *F* induces an isomorphism from *ℋ*_*x*_^⊥^ onto *Fℋ*_*x*_^⊥^. Let *v*_*x*_ be the vector space of holomorphic normal vectors to *M* at *x*, or simply the holomorphic normal space of *M* at *x*; that is,

(13) $$\begin{array}{c} v_{x}=T_{x}^{⊥}M\cap JT_{x}^{⊥}M. \end{array}$$

Then *v*_*x*_ defines a differentiable vector subbundle of *T* ^⊥^ *M*. we have that

(14) $$\begin{array}{c} T^{⊥}M=FH^{⊥}⊕v,\;t\left(T^{⊥}M\right)=H^{⊥}, \end{array}$$

(15) $$\begin{array}{c} g\left(FH^{⊥},v\right)=0. \end{array}$$

## 3. Integrability

In this section we study the integrability of the holomorphic distribution *ℋ* and the purely real distribution *ℋ* ^⊥^. First we give the following.

**Lemma 4 .**

Let *M* be a generic submanifold in a nearly Kaehler manifold $\bar{M}$. Then

(16) $$\begin{array}{c} g\left(\sigma \left(X,JY\right)-\sigma \left(JX,Y\right),\eta \right)=g\left(2G{\left(X,Y\right)}^{⊥},\eta \right), \end{array}$$

for any vector *X*, *Y* ∈ *ℋ* and *η* ∈ *v*.

**Proof**

From (2) and (6), we obtain

(17) g(Jσ(X,U),η)=g(J∇¯UX,η)=g(∇¯UJX−G(U,X)⊥,η)=g(σ(JX,U)+G(X,U)⊥,η),

where *X* ∈ *ℋ*, *U* ∈ *TM*, and *η* ∈ *v*. This implies that

(18) $$\begin{array}{c} g\left(J\sigma \left(X,Y\right),\eta \right)=g\left(\sigma \left(JX,Y\right)+G{\left(X,Y\right)}^{⊥},\eta \right), \end{array}$$

where *X*, *Y* ∈ *ℋ* and *η* ∈ *v*. Since the second fundamental form *σ* is symmetric, we have

(19) $$\begin{array}{c} g\left(\sigma \left(JX,Y\right)+G{\left(X,Y\right)}^{⊥},\eta \right)=g\left(\sigma \left(JY,X\right)+G{\left(Y,X\right)}^{⊥},\eta \right). \end{array}$$

From the equations above, we prove the lemma.

**Proposition 5 .**

Let *M* be a generic submanifold in a nearly Kaehler manifold $\bar{M}$. Then the holomorphic distribution *ℋ* is integrable if and only if

(20) $$\begin{array}{c} g\left(\sigma \left(X,JY\right)-\sigma \left(JX,Y\right),FZ\right)=g\left(2G{\left(X,Y\right)}^{⊥},FZ\right), \end{array}$$

for any vector fields *X*, *Y* ∈ *ℋ*, and *Z* ∈ *ℋ* ^⊥^.

**Proof**

Since $\bar{M}$ is nearly Kaehlerian, using formulas (2) and (6), we have

(21) σ(X,JY)−σ(JX,Y) =∇¯XJY−∇XJY−∇¯YJX+∇YJX =G(X,Y)+J∇¯XY−G(Y,X)  −J∇¯YX+∇YJX−∇XJY =2G(X,Y)+J[X,Y]+∇YJX−∇XJY.

So we get

(22) σ(X,JY)−σ(JX,Y)−2G(X,Y) =J[X,Y]+∇YJX−∇XJY,

for any vector field *X*, *Y* in *ℋ*. If the holomorphic distribution *ℋ* is integrable, the right-hand-side of (22) lies in *TM*; thus we obtain *σ*(*X*, *JY*) − *σ*(*JX*, *Y*) = 2*G*(*X*,*Y*)^⊥^. In particular, we have (20). Conversely, if (20) holds, then by Lemma 4 and (14) we have *σ*(*X*, *JY*) − *σ*(*JX*, *Y*) = 2*G*(*X*,*Y*)^⊥^ for any vectors *X*, *Y* in *ℋ*. Thus by (22) we obtain *J*[*X*, *Y*] = ∇_*X*_ *JY* − ∇_*Y*_ *JX*. Since ∇_*X*_ *JY* − ∇_*Y*_ *JX* is tangent to *M*, this implies that [*X*, *Y*] lies in *ℋ*. Thus we proved the proposition from the Frobenius theorem.

**Proposition 6 .**

Let *M* be a generic submanifold in a nearly kaehler manifold *M*. Then the purely real distribution *ℋ* ^⊥^ is integrable if and only if

(23) $$\begin{array}{c} P\left\{A_{FW}Z-A_{FZ}W+\nabla_{W}PZ-\nabla_{Z}PW\right\}-2JG\left(W,Z\right) \end{array}$$

lies in *ℋ* ^⊥^, for any vector fields *Z*, *W* ∈ *ℋ* ^⊥^.

**Proof**

For any vector fields *Z*, *W* ∈ *ℋ* ^⊥^, (6) gives

(24) $$\begin{array}{c} J\left({\bar{\nabla }}_{Z}W\right)=J\nabla_{Z}W+J\sigma \left(Z,W\right), \end{array}$$

and it follows immediately from (6), (7), and (9) that

(25) J(∇¯ZW)=∇¯ZJW−G(Z,W)J(∇¯ZW)=∇¯ZPW+∇¯ZFW−G(Z,W)J(∇¯ZW)=∇ZPW+σ(Z,PW)−AFWZJ(∇¯ZW) +DZFW−G(Z,W).

From which we obtain

(26) −∇ZW−σ(Z,W)=J∇ZPW+Jσ(Z,PW) −JAFWZ+JDZFW−JG(Z,W).

Comparing the tangential parts, we have

(27) ∇ZW=−P∇ZPW−tσ(Z,PW) +PAFWZ−tDZFW−G(Z,JW)⊤.

Thus we get

(28) [Z,W]=P{AFWZ−AFZW+∇WPZ−∇ZPW}[Z,W] +t{σ(W,PZ)−σ(Z,PW)+DWFZ−DZFW}[Z,W] −2JG(W,Z).  

Since *t*(*T* ^⊥^ *M*) = *ℋ* ^⊥^, this implies that [*Z*, *W*] lies in *ℋ* ^⊥^ if and only if (23) lies in *ℋ* ^⊥^. The proposition is proved.

**Theorem 7 .**

Let *M* be a generic submanifold in a nearly Kaehler manifold $\bar{M}$. If *ℋ* is integrable and its leaves are totally geodesic in *M*, then

(29) $$\begin{array}{c} g\left(\left(G+J\sigma \right)\left(H,H\right),H^{⊥}\right)=0. \end{array}$$

**Proof**

Since *ℋ* is integrable and its leaves are totally geodesic in *M*, we have ∇_*X*_ *Y* ∈ *ℋ*, for any *X*, *Y* ∈ *ℋ*. So *g*(∇_*X*_ *Z*, *Y*) = *g*(∇_*X*_ *Y*, *Z*) = 0, and we can get ∇_*X*_ *Z* ∈ *ℋ* ^⊥^ for any vector *X* ∈ *ℋ* and *Z* ∈ *ℋ* ^⊥^. From (2), (6), (7), and (9), we get

(30) g(∇XZ,JY) =−g(J∇¯XZ,Y) =g(G(X,Z)−∇¯XJZ,Y) =g(G(X,Z),Y)−g(∇¯XPZ,Y)−g(∇¯XFZ,Y) =g(G(X,Z),Y)−g(∇XPZ,Y)+g(AFZX,Y),

which implies that

(31) −g(G(X,Y),Z)+g(σ(X,Y),JZ)  =−g(G(X,Y)+Jσ(X,Y),Z)=0.

This proves the theorem.

**Theorem 8 .**

Let *M* be a generic submanifold in a nearly Kaehler manifold $\bar{M}$. If *ℋ* ^⊥^ is integrable and its leaves are totally geodesic in *M*, then

(32) $$\begin{array}{c} g\left(\left(G-J\sigma \right)\left(H,H^{⊥}\right),H^{⊥}\right)=0. \end{array}$$

**Proof**

Under the hypothesis, for any vector fields *X* in *ℋ* and *Z*, *W* in *ℋ* ^⊥^, it follows from (3), (6), (7), and (9) that

(33) g(∇ZX,W)=g(J∇¯ZX,JW)=g(−G(Z,X)+∇¯ZJX,PW+FW)=g(G(X,Z),JW)+g(∇¯ZJX,PW) +g(∇¯ZJX,FW)=g(G(X,Z),JW)+g(σ(JX,Z),FW)=−g(JG(X,Z)+Jσ(JX,Z),W)=g(G(JX,Z)−Jσ(JX,Z),W).

That is,

(34) $$\begin{array}{c} g\left(G\left(JX,Z\right)-J\sigma \left(JX,Z\right),W\right)=0. \end{array}$$

From this we obtain the theorem.

## 4. Generic Submanifolds with Parallel Canonical Structure

For the endomorphism *P* : *TM* → *TM*, we put

(35) $$\begin{array}{c} \left({\bar{\nabla }}_{X}P\right)Y=\nabla_{X}PY-P\nabla_{X}Y, \end{array}$$

for any vector fields *X*, *Y* ∈ *TM*. The endomorphism *P* is said to be parallel if ${\bar{\nabla }}_{X}P=0$ for any vector *X* ∈ *TM*. From (6), (7), and (9) we can obtain the following:

(36) J∇XY+Jσ(X,Y)=J∇¯XYJ∇XY+Jσ(X,Y)=∇¯XJY−G(X,Y)J∇XY+Jσ(X,Y)=∇¯XPY+∇¯XFY−G(X,Y)J∇XY+Jσ(X,Y)=∇XPY+σ(X,PY)J∇XY+Jσ(X,Y) −AFYX+DXFY−G(X,Y).

That is,

(37) P∇XY+F∇XY+tσ(X,Y)+fσ(X,Y) =∇XPY+σ(X,PY)−AFYX+DXFY−G(X,Y).

By comparing the tagential parts, we have the following:

(38) $$\begin{array}{c} \left({\bar{\nabla }}_{X}P\right)Y=t\sigma \left(X,Y\right)+A_{FY}X+G{\left(X,Y\right)}^{⊤}. \end{array}$$

Therefore, for any vector fields *X*, *Y*, *Z* ∈ *TM*, we have

(39) g((∇¯XP)Y,Z)=g(tσ(X,Y)+AFYX+G(X,Y)⊤,Z)g((∇¯XP)Y,Z)=−g(σ(X,Y),JZ)+g(AFYX,Z)g((∇¯XP)111,Z)+g(G(X,Y)⊤,Z)g((∇¯XP)Y,Z)=g(AFYZ−AFZY+G(Y,Z)⊤,X).

So, we obtain the Lemma as follows.

**Lemma 9 .**

Let *M* be a generic submanifold in a nearly Kaehler manifold $\bar{M}$. The P is parallel, that is, $\bar{\nabla }P=0$, if and only if

(40) $$\begin{array}{c} G{\left(U,V\right)}^{⊤}=A_{FV}U-A_{FU}V, \end{array}$$

for any vectors *U*, *V* ∈ *TM*.

**Theorem 10 .**

Let *M* be a generic submanifold in a nearly Kaehler manifold $\bar{M}$. If *P* is parallel, then

(i) *G*(*U*,*X*)^*⊤*^ = −*A*_*FU*_ *X*, for any vector fields *X* ∈ *ℋ* and *U* ∈ *TM*
(ii) the holomorphic distribution *ℋ* is intergrable.

**Proof**

From Lemma 9, for any vector fields *X* ∈ *ℋ* and *U* ∈ *TM*, we know *FX* = 0; this implies that *G*(*U*,*X*)^*⊤*^ = −*A*_*FU*_ *X*. On the other hand, for any vector fields *X*, *Y* ∈ *ℋ*, *G*(*X*,*Y*)^*⊤*^ = −*A*_*FX*_ *Y* = 0, then *G*(*X*, *Y*) is normal to *M*. By (i), we can get

(41) $$\begin{array}{c} g\left(G\left(U,X\right),Y\right)=g\left(-A_{FU}X,Y\right); \end{array}$$

that is,

(42) $$\begin{array}{c} g\left(G\left(X,Y\right)-J\sigma \left(X,Y\right),U\right)=0, \end{array}$$

for any vector fields *X*, *Y* ∈ *ℋ* and *U* ∈ *TM*. The equations above imply that

(43) $$\begin{array}{c} g\left(\sigma \left(X,Y\right),FZ\right)=0, \end{array}$$

for any vector fields *X*, *Y* ∈ *ℋ* and *Z* ∈ *ℋ* ^⊥^. These give

(44) $$\begin{array}{c} g\left(\sigma \left(X,JY\right)-\sigma \left(JX,Y\right),FZ\right)=g\left(2G{\left(X,Y\right)}^{⊥},FZ\right). \end{array}$$

From Proposition 5, the theorem holds.

For the normal bundle-valued 1-form *F*, we put

(45) $$\begin{array}{c} \left({\bar{\nabla }}_{X}F\right)Y=\nabla_{X}FY-F\nabla_{X}Y. \end{array}$$

For any vector fields *X*, *Y* ∈ *TM*. The endomorphism *F* is said to be parallel if ${\bar{\nabla }}_{X}F=0$ for any vector *X* ∈ *TM*. By comparing the normal parts of (37), we have the following:

(46) $$\begin{array}{c} \left({\bar{\nabla }}_{X}F\right)Y=f\sigma \left(X,Y\right)-\sigma \left(X,PY\right)+G{\left(X,Y\right)}^{⊥}, \end{array}$$

for any vectors *X*, *Y* ∈ *TM*. Hence, for any vector field *ξ* ∈ *T* ^⊥^ *M*, it follows from (4), (8), and (10) that

(47) g((∇¯XF)Y,ξ) =g(fσ(X,Y)−σ(X,PY)+G(X,Y)⊥,ξ) =g(Jσ(X,Y),ξ)−g(σ(X,PY),ξ)+g(G(X,Y),ξ) =−g(σ(X,Y),fξ)−g(σ(X,PY),ξ)+g(G(X,Y),ξ) =−g(AfξY+AξPY−G(Y,ξ)⊤,X).

From which we obtain the Lemma as follows.

**Lemma 11 .**

Let *M* be a generic submanifold in a nearly Kaehler manifold $\bar{M}$. The *F* is parallel, that is, $\bar{\nabla }F=0$, if and only if

(48) $$\begin{array}{c} A_{f\xi }X+A_{\xi }PX=G{\left(X,\xi \right)}^{⊤}, \end{array}$$

for any vectors *X* ∈ *TM* and *ξ* ∈ *T* ^⊥^ *M*.

**Theorem 12 .**

Let *M* be a generic submanifold in a nearly Kaehler manifold $\bar{M}$. If *F* is parallel, then the holomorphic distribution *ℋ* is intergrable.

**Proof**

From (46) we have,

(49) g((∇¯XF)Y,ξ) =g(fσ(X,Y)−σ(X,PY)+G(X,Y)⊥,ξ) =g(Jσ(X,Y),ξ)−g(σ(X,PY),ξ)  +g(G(X,Y)⊥,ξ),

for any vectors *X*, *Y* ∈ *ℋ* and *ξ* ∈ *T* ^⊥^ *M*. Since *F* is parallel, then

(50) $$\begin{array}{c} g\left(J\sigma \left(X,Y\right),\xi \right)-g\left(\sigma \left(X,PY\right),\xi \right)+g\left(G{\left(X,Y\right)}^{⊥},\xi \right)=0. \end{array}$$

That is,

(51) $$\begin{array}{c} J\sigma \left(X,Y\right)=\sigma \left(X,JY\right)-G{\left(X,Y\right)}^{⊥}. \end{array}$$

This implies that

(52) $$\begin{array}{c} g\left(\sigma \left(X,JY\right)-\sigma \left(JX,Y\right),FZ\right)=g\left(2G{\left(X,Y\right)}^{⊥},FZ\right), \end{array}$$

for any vectors *X*, *Y* ∈ *ℋ* and *Z* ∈ *ℋ* ^⊥^. From Proposition 5, the theorem holds.
